# Standardized angiographic workflow and technique for genicular artery embolization

**DOI:** 10.1186/s42155-026-00694-8

**Published:** 2026-05-01

**Authors:** Seyed A. Astani, Amanda Oliveira, Brandon Matty, Jafar Golzarian

**Affiliations:** 1 North Star Vascular & Interventional, Golden Valley, MN USA; 2 GE HealthCare, Chicago, USA

**Keywords:** Genicular artery embolization, Knee osteoarthritis, CBCT, Interventional radiology, Embolization technique

## Abstract

Genicular artery embolization (GAE) has emerged as a safe and encouraging minimally invasive treatment option for patients with chronic knee osteoarthritis (OA) who have failed conventional therapies. This technical report describes a standardized imaging and embolization protocol developed at a single institution, based on over 250 procedures. The technique integrates sheathless femoral access, routine use of cone-beam computed tomography (CBCT) with advanced embolization planning and guidance software, and a selective embolization strategy using resorbable or permanent agents. Procedures are performed in an outpatient setting, with same-day discharge and routine post-procedural physical therapy. The method described emphasizes safety, reproducibility, and precision, and provides a potential framework for broader standardization in GAE.

## Introduction

Genicular artery embolization (GAE) is a minimally invasive treatment for chronic knee osteoarthritis (OA) offered to patients who have failed conservative therapy, are not surgical candidates for total knee arthroplasty (TKA), or prefer to avoid surgery. Although published studies demonstrate safety and effectiveness, significant procedural variability persists, reflecting the limited technical literature addressing workflow standardization and the integration of modern technologies that may enhance procedural efficiency and optimization [1].

Although early GAE approaches targeted only the most symptomatic compartments [2, 3], recent studies support embolizing all genicular arteries demonstrating abnormal neovascularization [4–6]. However, comprehensive embolization can be technically challenging due to difficulty visualizing vessel origin.

This technical note summarizes our experience with over 250 GAEs performed in 12 months by two board-certified interventional radiologists (31- and 9-years post-fellowship). We describe a workflow using routine cone-beam CT (CBCT) coupled with advanced AI-based planning and guidance software. This protocol facilitates vessel access, and treatment of the abnormal vessels.

### Patient evaluation

Patients with symptomatic knee OA and imaging within the previous 12 months are evaluated by a qualified clinician. If symptoms have changed, updated weight-bearing radiographs are obtained. MRI is used for atypical or nonspecific presentations to exclude alternative causes such as meniscal tears or ligamentous injury. Contributing factors—peripheral arterial disease, venous insufficiency, or referred pain—are evaluated but not considered exclusion criteria. Additional imaging is obtained as needed to confirm OA as the primary pain generator. WOMAC and VAS scores are recorded at baseline and in different timepoints.

### Procedure setting and sedation

GAE is performed in an outpatient-based laboratory using an angiographic suite (Allia IGS740, GE HealthCare, Chicago, IL). Procedures use local anesthesia with moderate sedation available with most patients electing sedation. Vital signs are continuously monitored.

### Technique

#### Vascular access

Antegrade access to the superficial femoral artery (SFA) is obtained under real-time ultrasound guidance using a micropuncture set. Proper access technique is critical; a 45 degree oblique puncture angle facilitates catheter advancement, reduces resistance, and minimizes vessel stress. Ipsilateral SFA is preferred to the ipsilateral common femoral puncture (CFA). CFA puncture sites often lie near the inguinal crease, therefore limiting the working space and room for catheterization. Another limitation is the fact that wire preferentially enters the profunda femoralis artery causing technical difficulties. The contralateral CFA is a good option and is a good alternative in obese patients.

A 0.035-inch Amplatz Super Stiff wire (Boston Scientific, Marlborough, MA, USA) is advanced, allowing direct placement of the base catheter without a vascular sheath, minimizing access diameter. A 4 F Cordis TEMPO™ Berenstein catheter (65 cm, 90° angled tip) (Cordis, Santa Clara, CA, USA) is used.

Conversion to a sheath is performed for access-site oozing, hematoma, or suboptimal puncture angle, with placement of a 5 F sheath (Terumo Medical, Somerset, NJ USA). In anticoagulated patients, sheath placement may be performed proactively to ensure reliable hemostasis.

#### Initial imaging and CBCT acquisition

The base catheter is positioned in the mid-to-proximal thigh, proximal to the origin of the descending genicular artery (DGA). An anteroposterior DSA is performed using 60% diluted iodinated contrast (Visipaque™ 320, GE HealthCare, Princeton, NJ, USA). A 5-s, 200-degree CBCT acquisition follows, with an injection rate of 2.5–4 mL/sec for 9 s (22.5–36 mL total) and a standard delay of 4 s. Delay is extended (5–6 s) when reduced cardiac output or delayed perfusion is identified.

#### Vessel segmentation and planning

**Fig. 1:**
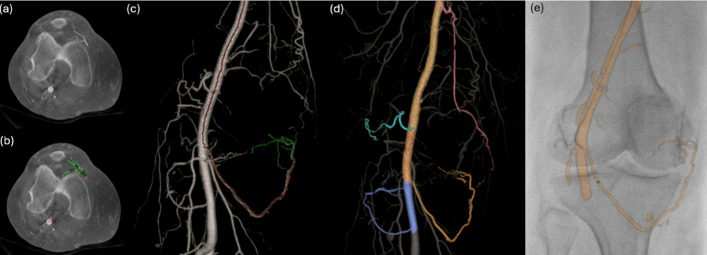
Advanced planning on CBCT using Virtual Injection technology (Embo ASSIST AI, GE HealthCare) **a** Contrasted CBCT with a 10-mm MIP axial view demonstrates initial opacification of the genicular branches from a superficial femoral artery injection. **b** Virtual Injection planning. A single-click selection of the injection point automatically highlights the target artery feeder (in green) and the catheter path (in red). **c** Zero-click automatic vessel segmentation generates a 3D vascular model displaying the full arterial tree, including the highlighting target artery feeder (in green) and catheter path (in red). **d** Segmented model with selected genicular arteries, including the descending genicular artery (red), superior lateral artery (cyan), inferior lateral (blue), and inferior medial artery (orange). **e** Automatic 3D model overlay onto live fluoroscopy provides augmented guidance for catheter navigation and embolization

**Fig. 2:**
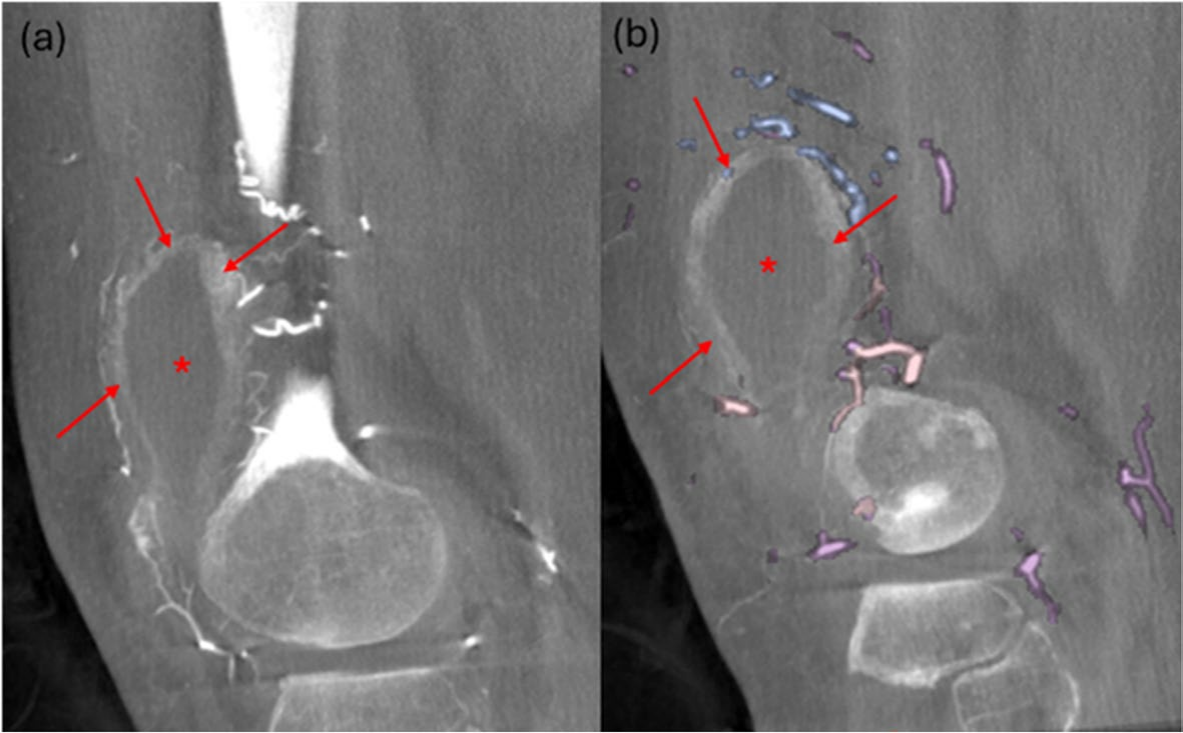
**a** Sagittal view from contrasted CBCT with injection from Superficial Femoral Artery (SFA) demonstrating joint effusion (*), synovial thickening (red arrows) and hyperemia. **b** Sagittal view with Embo ASSIST AI vessel segmentation overlay highlighting vessels supplying the pathological area. Arrows indicate synovial thickening, and * denotes joint effusion

CBCT datasets are processed using Embo ASSIST AI (Virtual Injection and vessel segmentation software) (GE HealthCare, Chicago, IL, USA), which automatically segments arteries, highlights targets, and simulates virtual injection paths (Fig. 1). Multiplanar CBCT allows detailed evaluation of patellar and synovial branches. Combined with planning software, the operator identifies target genicular arteries, visualizes anastomoses, and assesses dominant flow patterns (Fig. 2).

Using the segmented 3D arterial model, all arteries supplying the joint are assessed and embolization is not restricted to one vessel per compartment. Any vessel showing hyperemia or synovial supply is considered a target. Virtual selection of embolization points and review of backflow zones allow anticipation of embolic distribution and reduce non-target risk.

When using Emboassist, the 3D roadmap overlays live fluoroscopy and remains registered despite table or gantry movement. If misalignment occurs due to patient motion, the overlay is corrected at tableside using DSA without repeating CBCT.

#### Microcatheterization

A 1.7F (ID 0.40 mm) to 2.1F (ID 0.46 mm) angled-tip microcatheter (Merit Maestro®, Pursue®, Asahi Veloute®) is introduced through the base catheter. The angled design improves selective catheterization, especially with a stiff base catheter. Smaller 1.7F microcatheters improve distal access but may reduce pushability and torque response. A microwire is often unnecessary, streamlining the procedure and reducing fluoroscopy time.

Embo ASSIST AI assists with orienting the base catheter toward branch ostia. When aligned in profile, microcatheter advancement is frequently successful on the first attempt. This precision improves efficiency, minimizes vessel trauma, reduces angiograms, and enhances operator confidence.

#### Embolization strategy

**Fig. 3:**
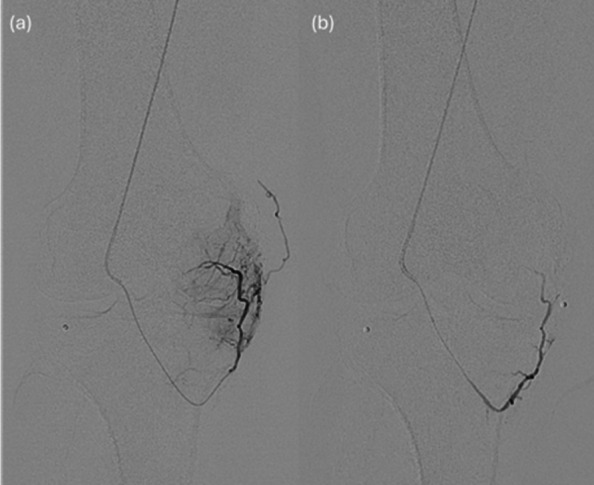
Hand injected DSA images of inferior medial genicular artery (**a**) showing blush pre-embolization and (**b**) post embolization

Embolization is not compartment-based; instead, all arteries contributing to pathological perfusion or hyperemia are treated. A proximal DSA identifies collateral takeoff. If no blush is seen, the microcatheter is advanced distally beyond branches supplying skin or muscle, and a limited DSA is performed to detect synovial blush and collateral pathways (Fig. 3).

The primary embolic agent is resorbable imipenem/cilastatin (500 mg in 10 mL contrast) (Fresenius Kabi, Lake Zurich, IL, USA), used when non-permanent embolization is indicated. When permanent embolization is required, 100–300 µm spherical particles, typically Embosphere® (Merit Medical, South Jordan, UT, USA), are used, with particle size being the key factor.

We use resorbable particles based on favorable safety data and prior studies demonstrating comparable technical and clinical outcomes [1, 7]. Escalation to permanent embolic particles is considered only in cases where persistent hyperemia remains after adequate embolization with resorbable material. Permanent particles are avoided when cutaneous or non-target vessels cannot be reliably excluded, to minimize the risk of non-target embolization. If there is concern about non-target embolization to the skin, preventive cold compresses reduce dermal perfusion intra-procedurally; if skin embolization is suspected afterward, warm compresses are applied to enhance perfusion.

#### Radiation optimization

Digital zoom at a collimated maximum field of view is used instead of magnification to reduce radiation exposure while maintaining image quality. Combined with software-guided targeting, this approach reduces repeated angiograms and decreases contrast and radiation dose.

#### Post-procedural care and follow-up

Access point hemostasis is achieved with 10 min of manual compression. In cases with a vascular sheath an Angio-Seal® (Terumo Medical, Somerset, NJ, USA) closer device is used. Patients recover for one hour and are discharged the same day. Strenuous activity is avoided for three days, and walking is encouraged. Pain typically improves within one week and continues over 1–3 months. Follow-up occurs at 3–4 weeks with physical therapy referral, then at 3, 6, and 12 months with WOMAC and VAS collection. Follow-up imaging was reserved for patients presenting with atypical symptoms or clinical failure.

#### Complications

Complications were classified according to the CIRSE classification system; only minor (grade 1–2) access-site events were observed in less than 2% of patients (3 out of 250 patients).

### Workflow advancements

Recent advancements in this workflow are centered on the integration of cone-beam CT (CBCT) with AI-assisted 3D-vessel segmentation. The resulting 3D model enables precise visualization of the genicular artery origin and course, which is essential for accurate target identification and embolization planning. Enhanced anatomical clarity allows identification of additional genicular and accessory vessels, facilitating faster and more efficient catheterization, reduced contrast use, greater procedural confidence, and optimized clinical outcomes.

### Procedure metrics

Among 251 patients treated in the past 12 months, the mean number of genicular arteries embolized was 5 ± 2.5 (range 2–11). Sheathless access was used in > 95% of cases, and the average embolic volume was 2.6 ± 0.9 mL for permanent particles and 5.0 ± 2.0 mL for resorbable imipenem (Table 1). Mean fluoroscopy time was 17.7 min; mean DAP was 10.7 Gy·cm^2^; air kerma was 54.1 mGy. Mean contrast volume was 64.4 mL (40–110 mL). Mean procedure time was 52 ± 18 min (20–109 min) (Table 2).

**Table 1:** Summary of procedural data (vessels embolized, amount of particles)

		Embolic agent (ml)	
Data spread	Number of vessels embolized	Sphere	Imipenem
Average, standard deviation	5(+/-2.5)	2.6(+/-0.9)	5.0(+/-2.0)
Range	[2-11]	[1.0-5.2]	[1.7-11.3]

**Table 2:** Summary of radiation dose and procedure time. Procedure time is defined as the period from either the puncture or timeout to when the provider exits the room

Data spread	Fluoroscopy time (min)	Air Kerma radiation dose (mGy)	Dose area product (Gy.cm2)	Procedure time (min)	Total contrast media (mL)
Average, standard deviation	17.7(+/-8.8)	54.1(+/-39.0)	10.7(+/-6.6)	52(+/-18)	64.4
Range	[1.6-59.2]	[11-261]	[208-52.1]	[20-109]	40-110ML

## Discussion

Vessel targeting in GAE may be guided by symptom-based or compartment-based localization or by angiographic identification of abnormal hypervascular blush [2, 8, 9]. Based on Landers' [13] study, embolization of abnormal vessels is associated with better outcome compared to the sham arm. In our experience, AI-assisted 3D visualization facilitated precise delineation of vessel origin and course, supporting confident multi-vessel embolization when indicated (mean 5 ± 2.5 vessels). Osteoarthritis-associated synovitis is increasingly recognized as a diffuse inflammatory process with pan-compartment neoangiogenesis [10–13], providing a biological rationale for treating all angiographically abnormal vessels when present.

## Conclusion

This report describes a structured, image-guided workflow for Genicular artery embolization incorporating cone-beam CT and AI-based planning software (Embo ASSIST AI). Based on over 250 cases, the technique emphasizes safety, vessel-specific targeting, and outpatient feasibility. While not designed as a comparative study, this workflow integrates contemporary interventional radiology technologies that may facilitate procedural consistency and anatomical precision. Further comparative and multicenter investigations are required to determine its impact on efficiency, radiation metrics, and clinical outcomes.

## Data Availability

The datasets generated and analyzed during the current study are not publicly available due to institutional policy and patient privacy protections but are available from the corresponding author upon reasonable request.

## Abbreviations

CBCT: Cone-beam computed tomography
DSA: Digital subtraction angiography
GAE: Genicular artery embolization
IRB: Institutional Review Board
MRI: Magnetic resonance imaging
NSVI: North Star Vascular & Interventional
OA: Osteoarthritis
SFA: Superficial femoral artery
TKA: Total knee arthroplasty
VAS: Visual Analog Scale
WOMAC: Western Ontario and McMaster Universities Osteoarthritis Index
